# Tailoring Community-Based Wellness Initiatives With Latent Class Analysis — Massachusetts Community Transformation Grant Projects

**DOI:** 10.5888/pcd11.130215

**Published:** 2014-02-13

**Authors:** Mariana Arcaya, Timothy Reardon, Joshua Vogel, Bonnie K. Andrews, Wenjun Li, Thomas Land

**Affiliations:** Author Affiliations: Timothy Reardon, Metropolitan Area Planning Council, Boston, Massachusetts; Joshua Vogel, Bonnie K. Andrews, Thomas Land, Massachusetts Department of Public Health, Boston, Massachusetts; Wenjun Li, University of Massachusetts Medical School, Worcester, Massachusetts.

## Abstract

**Introduction:**

Community-based approaches to preventing chronic diseases are attractive because of their broad reach and low costs, and as such, are integral components of health care reform efforts. Implementing community-based initiatives across Massachusetts’ municipalities presents both programmatic and evaluation challenges. For effective delivery and evaluation of the interventions, establishing a community typology that groups similar municipalities provides a balanced and cost-effective approach.

**Methods:**

Through a series of key informant interviews and exploratory data analysis, we identified 55 municipal-level indicators of 6 domains for the typology analysis. The domains were health behaviors and health outcomes, housing and land use, transportation, retail environment, socioeconomics, and demographic composition. A latent class analysis was used to identify 10 groups of municipalities based on similar patterns of municipal-level indicators across the domains.

**Results:**

Our model with 10 latent classes yielded excellent classification certainty (relative entropy = .995, minimum class probability for any class = .871), and differentiated distinct groups of municipalities based on health-relevant needs and resources. The classes differentiated healthy and racially and ethnically diverse urban areas from cities with similar population densities and diversity but worse health outcomes, affluent communities from lower-income rural communities, and mature suburban areas from rapidly suburbanizing communities with different healthy-living challenges.

**Conclusion:**

Latent class analysis is a tool that may aid in the planning, communication, and evaluation of community-based wellness initiatives such as Community Transformation Grants projects administrated by the Centers for Disease Control and Prevention.

## Introduction

Chronic diseases are among the leading causes of preventable death in the United States, accounting for roughly 75% of the nation’s health care costs (1). These diseases are related to tobacco use, physical inactivity, and poor diet. Community-based approaches to preventing chronic diseases are attractive because of their broad reach and low costs, especially relative to most medical interventions.

The Prevention and Public Health Fund established by the Patient Protection and Affordable Care Act of 2010 funds the Centers for Disease Control and Prevention (CDC) to administer the Community Transformation Grant (CTG) program. These grants fund community-level efforts to support Americans in healthy eating, active living, and tobacco-free living by using evidence-based strategies. In September 2011, CDC awarded approximately $103 million to 61 grantees (2), including the Massachusetts Department of Public Health (DPH), which received 2 CTG awards to fund chronic disease prevention efforts in 9 Massachusetts counties.

With CTG support, DPH has augmented ongoing tobacco control efforts and implemented quality-improvement coaching in select federally qualified community health centers. Most of the grant, however, has been used to expand an existing municipal-level initiative called Mass in Motion. The initiative, which focuses on preventing overweight and obesity by promoting healthful eating and physical activity (3), serves 52 of Massachusetts’ 351 cities and towns.

Expanding Mass in Motion to more municipalities creates implementation and evaluation challenges. To be successful, municipal efforts should be tailored to community context, particularly sociodemographic composition, pressing health challenges, and health-related community resources. Given the complexity and interaction of these factors, there is no “one size fits all” for successful intervention strategies (4). However, the high number of Massachusetts municipalities precludes cost-effective creation of tailor-made approaches to prevention in each community. Furthermore, because many communities share similar challenges, a mechanism is needed to facilitate sharing of successes and best practices among peer communities.

A data-driven classification system that groups municipalities, the level at which Mass in Motion generally operates, according to relevant characteristics balances these concerns, supporting a tailored-yet-efficient approach to program implementation and evaluation. DPH, in partnership with the Metropolitan Area Planning Council and the University of Massachusetts Medical School, designed an empirically based “Prevention and Wellness Community Typology” using latent class analysis (LCA) that classifies municipalities into distinct groups based on prevention needs, community assets, and other contextual information. These groupings help DPH customize communications messages, set realistic goals for intervention outcomes, and evaluate the success of community-based wellness initiatives.

LCA is a statistical method for identifying underlying groups of similar individuals or units from a heterogeneous sample. LCA methods have been applied to study mental health problems (5,6), substance use patterns (7,8), skin cancer risk (9), back pain symptoms (10), and obesity-related health behaviors (11–13). However, we found only 1 paper that applied LCA to categorize US communities or neighborhoods into general archetypes (14). In this report, we show a novel application of LCA models to improve and better understand a suite of ongoing community-based public health interventions supported by CDC.

## Methods

After key informant interviews with regional planners and public health practitioners, literature reviews, and exploratory data analyses, we identified 6 domains of municipal-level characteristics expected to affect the implementation or evaluation of community-based prevention strategies in Massachusetts. These domains are composed of 1) health behaviors or outcomes relevant to program goals, 2) housing and land use characteristics, 3) transportation patterns, 4) retail environment, 5) socioeconomics, and 6) demographic composition. These domains were used only in framing the selection input indicators a priori, not in representing distinct latent variables for which separate class solutions would be estimated.

The domain descriptions are as follows: Domain 1 (health behaviors and outcomes) captures baseline metrics that the CTG program seeks to improve (eg, fruit and vegetable intake). Domains 2 through 4 include local conditions that are involved in community-based interventions. For example, domain 2 (housing and land use) includes an indicator of subsidized housing inventory because communities are working to promote tobacco-free living in this setting. Similarly, domain 3 (transportation patterns) includes measures relevant to the walking environment. Domain 4 (retail environment) includes counts and densities of local business establishments with which many initiatives may require collaborations. Domains 5 (socioeconomics) and 6 (demographics) are expected to affect the way interventions work across communities. Domain 5 includes conditions such as median household income, which can affect the ability of residents to change some of their health behaviors. Domain 6 focuses on demographic composition because interventions may work differently among different subpopulations. For example, responses to active living and healthy eating interventions may vary by age.

We selected 55 variables to represent the 6 domains. The Appendix provides detailed metadata on each variable. Briefly, the sources and types of data were as follows:

- Massachusetts Behavioral Risk Factor Surveillance System (BRFSS) (2009): community-level prevalence estimates for selected obesity-related risk outcomes and behaviors, including diabetes, hypertension, current smoking, obesity, fruits and vegetable consumption, and physical activity among adults. Municipal-level BRFSS estimates are constructed by using a small-area estimation method (15,16) that weights data according to age, sex, race/ethnicity, and poverty rates.
- Hospital discharge data from the Massachusetts Center for Health Information and Analysis (2010): annual rates of hospitalizations for obesity-related health outcomes for 1) hypertension and hypertensive diseases, 2) transient ischemic attack, 3) major cardiovascular disease, 4) heart disease, and 5) cerebrovascular disease. We include both unadjusted and age-adjusted rates because the 2 indicators provide different information. The former indicates the magnitude of disease burden in communities while the latter highlights geographic disparities by allowing for the comparison of municipalities after removing age as a determinant of hospitalization.
- Massachusetts Registry of Vital Records (2009): percentage of births to smoking mothers.
- Massachusetts Tobacco Cessation and Prevention Program surveillance data (fiscal year 2013): count of tobacco and alcohol retail outlets.
- Massachusetts Department of Public Health (2011): percentage of overweight or obese students in grade 1 and grade 10.
- Massachusetts Board of Registration in Medicine directory (2009): number of practicing physicians.
- US Census (2000 and 2010), and American Community Survey (2006–2010): indicators of demographic composition (age structure and racial/ethnic composition), socioeconomic status (median household income, poverty, and unemployment rates), use of automobiles for commuting to work, housing stock composition, and population change.
- Massachusetts Executive Office of Energy and Environmental Affairs: air-photo–based land use data, 1970 and 1999; measures of how much land is developed or vacant and developable as of 2001; and estimates of vehicle miles traveled (VMT) per household.
- Massachusetts Department of Housing and Community Development (2012): Subsidized Housing Inventory, a measure of publically subsidized and deed-restricted affordable housing units in each municipality, expressed as a percentage of a community’s year-round housing units.
- InfoGroup (2011): counts of business establishments.

We compiled data for all 351 municipalities for each indicator, except where estimates were deemed unstable by DPH, in which case data were coded as missing. We ranked municipalities according to each of these indicators and assigned the municipality’s decile for each indicator in our model. For example, Boston, Massachusetts’ most populous municipality, was assigned a value of 10 for its 2010 population value rather than 617,594, the actual number of residents. We used this approach to prioritize relative similarity above absolute similarity so that municipalities with particularly extreme values on multiple indicators would not end up in classes by themselves while the bulk of communities were grouped into 1 large class. Roughly equal-sized groups provide functional peer groups for communities and make stratified sampling by class possible. Deciles did a better job than raw values did of operationalizing this idea of relative similarity. The resulting data set was a matrix of 351 municipalities by 55 indicators, populated with decile values for each municipality-indicator cell.

LCA, also known as a finite mixture modeling, allows researchers to detect underlying (latent) subgroups from observable variables. Subgroups are identified that produce independence among the observed variables conditional on class membership such that variables that are usually highly correlated, such as median household income and housing unit density, would no longer be correlated within each class.

Because the goal of this analysis was to help program staff tailor intervention approaches to community needs and context, we constrained our model to a 10-class solution, which was thought to be the highest number of classes tolerable from a program planning and evaluation perspective. Because observed variables (eg, population size) predicted missing data patterns, we were able to use full-information maximum likelihood estimation methods to predict the probabilities of each community belonging to each latent class. Each municipality was assigned to the latent class that the municipality had the highest probability of belonging to. The analysis was conducted using MPlus version 6.1 (Muthén and Muthén, Los Angeles, California).

## Results

Health-relevant community characteristics varied widely among Massachusetts municipalities (Table 1), highlighting the need to tailor community prevention efforts. For example, municipal smoking prevalence ranged from 4.7% to 29.2%, and the percentage of residents ever diagnosed with hypertension ranged from 10.3% to 34.5%. Sociodemographic composition and environmental characteristics also varied widely. The percentage of the population that was non-Hispanic white in 2010 ranged from roughly 20% to 98% at the municipal level while the percentage of housing that is high density or multi-family ranged from 0% to 100%.

**Table 1:** Community Characteristics by Latent Class in Analysis of Massachusetts Communities

Indicator	Class 1, Mean (SD)	Class 2, Mean (SD)	Class 3, Mean (SD)	Class 4, Mean (SD)	Class 5, Mean (SD)	Class 6, Mean (SD)	Class 7, Mean (SD)	Class 8, Mean (SD)	Class 9, Mean (SD)	Class 10, Mean (SD)	State Mean (SD)	State Minimum	State Maximum
**Domain 1: health behaviors and outcomes**													
Current smokers, % adults	16.1 (3.4)	20.8 (2.2)	14.6 (2.5)	18.3 (2.4)	10.7 (3.7)	20.2 (3.0)	13.0 (3.7)	15.6 (2.2)	10.6 (4.0)	21.3 (3.4)	16.5 (4.8)	4.7	29.2
Consumed recommended no. of fruit and vegetable servings, % adults	27.3 (2.6)	22.5 (1.3)	25.4 (2.1)	22.9 (1.4)	28.1 (2.9)	21.3 (1.5)	25.5 (2.4)	23.4 (1.4)	27.5 (2.7)	20.2 (1.6)	24.2 (3.3)	16.7	34.2
No exercise in past 30 days, % adults	14.7 (2.4)	21.4 (2.6)	18.3 (2.7)	19.5 (2.1)	13.1 (2.5)	23.4 (3.1)	16.4 (2.3)	19.3 (1.6)	14.6 (2.5)	27.6 (5.5)	19.1 (5.2)	8.9	40.6
Diabetes, %	4.2 (0.6)	5.1 (0.4)	5.4 (1.1)	4.4 (0.4)	3.5 (0.5)	5.7 (0.8)	4.0 (0.4)	4.6 (0.4)	3.5 (0.8)	6.4 (1.3)	4.7 (1.1)	1.4	9.3
Hypertension, %	21.4 (2.4)	24.9 (1.9)	26.3 (4.0)	22.1 (1.7)	19.0 (1.9)	26.0 (2.7)	20.6 (1.8)	22.0 (1.2)	18.4 (3.3)	25.2 (2.5)	22.7 (3.6)	10.3	34.5
Obese adults (%)	26.0 (3.2)	29.8 (1.6)	25.2 (3.2)	27.9 (1.5)	20.5 (3.5)	29.0 (2.3)	23.1 (2.9)	25.3 (1.6)	18.6 (3.2)	30.3 (2.8)	25.9 (4.4)	12.7	34.5
Births to smokers, per 1,000 births	11.7 (6.3)	14.6 (4.7)	7.8 (2.8)	7.9 (3.4)	2.8 (1.3)	12.6 (6.0)	3.5 (1.8)	4.9 (1.6)	2.2 (1.7)	9.5 (4.8)	8.0 (5.6)	0.4	33.5
Overweight or obese 1st graders, %	25.6 (7.7)	29.2 (4.1)	23.0 (7.3)	25.7 (8.1)	16.0 (5.3)	32.3 (6.7)	21.3 (5.1)	29.3 (6.3)	22.3 (7.6)	33.7 (4.7)	26.1 (8.2)	3.5	46.0
Overweight or obese 10th graders, %	30.7 (5.1)	25.1 (10.4)	27.3 (8.9)	27.7 (7.0)	22.3 (3.5)	36.7 (6.7)	25.7 (4.3)	31.9 (10.1)	28.3 (11.0)	35.6 (7.5)	29.6 (8.5)	3.3	60.3
Cardiovascular disease hospitalizations, n per 100,000 population	1,001.7 (544.9)	1,417.8 (507.1)	1,895.8 (412.6)	1,380.6 (262.8)	1,188.5 (320.2)	1,925.4 (408.1)	1,452.5 (332.3)	1,945.1 (298.0)	1,375.0 (327.0)	1,799.6 (318.3)	1,526.6 (495.0)	0	3,050.3
Cerebrovascular hospitalizations, n per 100,000 population	12.8 (52.6)	232.1 (159.4)	368.7 (98.2)	210.1 (62.0)	221.1 (61.6)	340.0 (82.0)	226.0 (77.2)	313.1 (61.8)	233.6 (57.2)	284.1 (52.1)	256.6 (110.9)	0	584.1
Heart disease hospitalizations, n per 100,000 population	733.3 (522.2)	1,082.9 (370.7)	1,352.4 (338.9)	1,049.0 (210.2)	889.5 (278.2)	1,405.1 (293.1)	1,098.3 (253.0)	1,456.1 (222.1)	1,015.8 (258.1)	1,331.1 (252.5)	1,138.2 (379.0)	0	2,273.0
Hypertension (and related) hospitalizations, n per 100,000 population	0	0	0	9.8 (27.6)	8.2 (25.9)	68.4 (40.2)	45.9 (17.7)	55.4 (21.1)	41.5 (7.2)	67.7 (27.8)	33.0 (36.9)	0	184.1
Stroke hospitalizations, n per 100,000 population	83.1 (149.0)	397.3 (225.5)	460.4 (171.0)	410.5 (97.2)	286.4 (97.8)	508.2 (123.7)	378.2 (104.2)	493.4 (82.5)	292.4 (87.6)	427.7 (106.6)	395.2 (159.0)	0	973.5
Age-adjusted cardiovascular disease hospitalizations, n per 100,000 population	1,025.3 (634.0)	1,389.1 (487.5)	1,214.3 (235.8)	1,549.6 (272.6)	1,382.5 (352.7)	1,567.5 (382.2)	1,473.6 (279.2)	1,613.5 (181.7)	1,097.2 (190.4)	1,613.0 (252.2)	1,410.0 (411.3)	0	2,927.1
Age-adjusted cerebrovascular hospitalizations, n per 100,000 population	10.9 (44.8)	236.0 (189.6)	236.0 (78.3)	235.7 (75.6)	249.2 (85.8)	274.4 (71.4)	228.6 (64.2)	261.2 (55.6)	186.4 (34.3)	255.2 (52.7)	230.1 (94.8)	0	538.9
Age-adjusted heart disease hospitalizations, n per 100,000 population	746.9 (589.4)	1,060.3 (327.5)	864.5 (185.4)	1,176.3 (227.5)	1,037.6 (312.4)	1,142.1 (285.7)	1,110.9 (211.1)	1,204.2 (118.9)	803.8 (150.5)	1,185.9 (184.6)	1,050.3 (328.5)	0	2,436.8
Age-adjusted hypertension (and related) hospitalizations, n per 100,000 population	0	0	0	11.3 (32.0)	6.3 (20.0)	58.8 (37.6)	44.0 (19.0)	46.1 (17.4)	36.4 (10.3)	64.0 (30.5)	29.7 (34.3)	0	171.3
Age-adjusted stroke hospitalizations, n per 100,000 population	80.2 (146.1)	355.2 (194.1)	307.4 (106.6)	448.9 (124.4)	330.6 (145.9)	418.1 (116.9)	380.0 (107.0)	410.5 (56.0)	237.1 (56.2)	393.1 (99.9)	361.8 (145.9)	0	963.9
**Domain 2: housing and land use**													
Population density, n per acre	0.1 (0.1)	0.1 (0.1)	0.8 (1.0)	0.5 (0.3)	0.7 (0.3)	1.4 (1.0)	1.4 (0.4)	3.2 (1.1)	7.9 (7.0)	7.6 (6.1)	1.9 (3.6)	0	29.3
Housing unit density, n units per residential acre	0.7 (0.2)	1.0 (0.2)	1.7 (0.7)	1.2 (0.3)	1.0 (0.3)	2.5 (1.2)	1.6 (0.3)	3.2 (1.0)	6.4 (6.3)	7.5 (5.1)	2.4 (3.1)	0.5	26.3
High-density housing, %	0.5 (1.8)	5.1 (5.6)	10.2 (9.8)	3.7 (4.3)	3.0 (3.4)	21.2 (15.6)	7.0 (5.3)	16.5 (11.7)	51.2 (33.7)	61.0 (29.6)	15.7 (23.8)	0	100.0
Medium-density housing, %	4.1 (4.2)	15.5 (8.9)	32.6 (14.4)	25.2 (12.5)	20.9 (13.0)	45.4 (16.6)	47.4 (13.9)	65.3 (13.7)	32.5 (25.5)	29.5 (23.6)	30.1 (22.0)	0	91.2
Low-density housing, %	95.4 (4.7)	79.4 (11.9)	57.2 (18.2)	71.1 (14.1)	76.1 (13.6)	33.4 (17.2)	45.6 (13.8)	18.2 (10.5)	16.3 (15.8)	9.4 (11.7)	54.2 (31.3)	0	100.0
Single-family homes, %	89.3 (9.0)	78.4 (10.8)	76.1 (13.0)	78.8 (11.0)	83.6 (11.9)	65.1 (14.6)	76.7 (11.4)	62.6 (10.0)	53.8 (24.7)	39.7 (15.5)	72.2 (19.2)	7.8	100.0
Developed land, %	6.6 (4.7)	7.9 (3.2)	29.5 (17.8)	19.1 (6.8)	28.5 (9.5)	32.8 (13.4)	39.9 (8.4)	58.0 (9.3)	72.2 (18.9)	62.7 (20.0)	32.0 (23.1)	1.2	99.1
Vacant developable land, %	53.1 (15.8)	54.7 (16.5)	29.5 (16.9)	54.4 (14.5)	31.8 (13.5)	37.1 (15.7)	26.6 (13.1)	15.5 (9.8)	10.2 (11.8)	16.7 (14.3)	36.0 (20.9)	0	100.0
Built-out land, %	11.3 (8.3)	13.3 (6.4)	49.9 (22.3)	27.1 (11.0)	48.5 (17.2)	47.6 (18.9)	61.5 (14.3)	79.1 (11.9)	84.0 (15.2)	76.6 (17.7)	45.5 (28.1)	2.6	100.0
Pre-1939 housing, %	28.8 (11.2)	33.8 (12.0)	28.7 (13.6)	21.9 (8.6)	21.1 (8.3)	29.9 (14.4)	17.1 (5.6)	26.4 (11.7)	43.1 (13.0)	40.0 (11.6)	28.2 (13.3)	1.0	63.9
Post-war housing, %	23.6 (9.0)	27.0 (8.3)	28.8 (7.3)	27.5 (9.2)	31.8 (10.3)	32.9 (8.9)	36.5 (10.7)	40.5 (7.0)	35.7 (7.8)	33.6 (7.6)	31.1 (10.0)	7.3	57.1
Post-1970 housing, %	47.6 (12.2)	39.2 (10.0)	42.5 (14.8)	50.6 (11.3)	47.2 (12.9)	37.3 (14.2)	46.4 (10.9)	33.2 (9.0)	21.1 (9.2)	26.4 (8.2)	40.8 (14.5)	7.8	80.8
Sewer-accessible housing, %	3.8 (8.2)	20.4 (23.8)	40.5 (33.5)	19.8 (18.1)	17.3 (20.0)	51.6 (31.8)	38.0 (25.7)	82.5 (24.1)	95.8 (4.4)	93.8 (8.9)	40.7 (37.2)	0	99.9
Commercial and industrial land use, %	1.9 (1.7)	4.4 (2.3)	6.8 (3.9)	5.3 (2.5)	3.8 (3.2)	10.2 (4.4)	9.1 (4.3)	16.1 (5.3)	9.9 (7.4)	16.5 (5.7)	7.8 (6.1)	0	35.0
Subsidized housing inventory, %	1.6 (4.2)	2.3 (2.7)	5.1 (2.4)	3.8 (1.9)	4.1 (2.7)	6.3 (3.0)	6.5 (3.0)	8.6 (2.2)	7.2 (3.2)	10.5 (3.7)	5.3 (4.0)	0	25.9
Residential land use change, %	81.9 (45.0)	57.9 (24.9)	57.7 (34.6)	95.4 (44.6)	74.4 (45.1)	49.0 (35.7)	65.8 (42.9)	25.4 (14.9)	10.7 (12.5)	17.6 (17.7)	58.8 (44.2)	−4.9	233.8
**Domain 3: transportation patterns**													
Daily vehicle miles traveled	80.5 (11.3)	75.4 (15.5)	61.6 (8.5)	87.3 (10.7)	83.7 (12.5)	63.6 (12.4)	74.6 (7.6)	59.4 (7.3)	46.8 (11.6)	44.6 (10.8)	70.1 (17.6)	20.9	111.3
Car/truck/van commuters, %	87.4 (7.1)	90.1 (13.2)	85.6 (6.2)	92.2 (3.3)	84.1 (6.9)	91.1 (7.1)	87.5 (4.8)	88.0 (4.3)	72.6 (14.6)	84.2 (11.1)	87.2 (9.3)	22.3	98.4
**Domain 4: retail environment**													
Businesses, n	50.2 (42.7)	76.2 (55.2)	390.9 (191.6)	283.2 (136.2)	301.3 (150.1)	760.8 (600.6)	847.2 (296.8)	1,606.4 (708.7)	1,796.3 (1,398.2)	3,097.3 (5,563.8)	806.4 (1,957.0)	3.0	33,149.0
Tobacco retailers, n	1.2 (1.1)	2.8 (1.8)	9.8 (5.5)	8.6 (4.5)	6.3 (4.4)	25.3 (14.2)	21.0 (9.5)	40.6 (18.9)	38.6 (38.5)	123.6 (151.0)	25.2 (58.2)	0.0	878.0
Tobacco retailers selling alcohol, n	0.7 (0.9)	1.7 (1.3)	1.5 (1.8)	2.1 (3.0)	1.7 (1.8)	4.9 (4.2)	3.3 (3.5)	3.5 (3.5)	5.8 (8.0)	15.0 (18.9)	3.8 (7.6)	0.0	102.0
Physicians, n	1.2 (3.6)	0.5 (1.2)	4.5 (4.8)	3.1 (3.3)	5.6 (7.5)	12.1 (12.4)	15.0 (11.9)	29.8 (23.4)	57.2 (64.3)	120.3 (339.4)	21.4 (109.8)	0.0	1984.0
**Domain 5: socioeconomics**													
Unemployment rate in 2000	2.3 (0.7)	3.2 (1.8)	3.1 (1.1)	2.7 (0.4)	2.0 (0.2)	3.3 (2.1)	2.2 (0.3)	2.3 (0.2)	2.0 (0.2)	3.2 (0.8)	2.7 (1.2)	0.8	17.4
Unemployment rate in 2010	6.6 (1.8)	9.3 (2.2)	8.3 (1.9)	8.9 (1.2)	6.6 (0.9)	9.9 (2.1)	7.3 (1.0)	7.4 (0.5)	6.1 (0.5)	10.1 (2.1)	8.1 (2.1)	3.1	22.3
Median annual household income, $	69,343.9 (13,059.3)	57,820.2 (9,365.0)	67,159.8 (13,080.6)	79,307.8 (8,452.0)	115,052.5 (22,524.9)	60,907.5 (11,301.5)	95,112.0 (15,087.7)	77,456.6 (9,161.2)	90,702.2 (24,093.1)	52,337.5 (11,561.0)	75,953.4 (23,163.3)	30,833.0	164,583.0
Poverty rate	6.3 (4.3)	7.1 (3.8)	6.8 (2.8)	5.1 (2.3)	3.5 (2.6)	7.8 (3.7)	3.8 (1.3)	5.5 (2.0)	7.5 (6.4)	14.5 (6.8)	6.7 (4.8)	0.0	31.7
**Domain 6: demographics**													
Non-Hispanic white, %	95.0 (5.9)	95.9 (1.0)	92.4 (4.1)	94.1 (3.5)	91.9 (4.8)	90.9 (5.1)	89.4 (5.8)	85.7 (7.3)	81.4 (9.0)	66.5 (19.9)	89.2 (11.3)	20.5	98.2
Non-Hispanic black, %	0.7 (0.6)	0.5 (0.4)	1.5 (1.2)	1.1 (1.5)	1.0 (1.2)	1.9 (1.8)	1.6 (1.3)	2.9 (2.5)	3.3 (3.4)	7.5 (8.5)	2.0 (3.5)	0.0	37.1
Non-Hispanic Asian, %	0.7 (0.6)	0.5 (0.4)	1.3 (1.1)	1.2 (0.8)	3.5 (3.4)	1.2 (0.8)	5.0 (4.6)	4.7 (2.9)	8.3 (4.7)	5.1 (5.8)	2.7 (3.7)	0.0	24.0
Latino/Hispanic, %	1.5 (0.8)	1.6 (0.7)	2.3 (1.3)	2.2 (1.6)	2.1 (1.3)	3.5 (3.9)	2.4 (1.5)	4.1 (3.0)	4.5 (3.1)	17.3 (16.8)	3.9 (7.0)	0.0	73.8
Aged <18 y, %	19.0 (3.1)	20.7 (2.5)	17.7 (3.6)	23.7 (1.9)	26.6 (3.5)	20.5 (3.1)	25.6 (2.4)	21.3 (1.6)	21.1 (5.8)	21.9 (3.0)	21.9 (4.1)	6.8	32.0
Aged ≥65 y, %	16.3 (5.3)	14.9 (2.9)	22.3 (6.7)	12.4 (2.4)	12.8 (2.9)	16.5 (4.2)	13.5 (3.1)	15.7 (1.9)	14.5 (3.0)	13.5 (2.6)	15.1 (4.5)	7.4	39.8
Total population in 2000	1,177.2 (718.4)	2,103.6 (1,133.9)	5,957.8 (2,799.7)	7,591.8 (2,848.2)	7,309.7 (2,886.1)	16,656.6 (9,420.7)	18,185.3 (5,833.7)	28,691.0 (12,096.2)	38,504.8 (23,708.0)	76,206.1 (98,391.4)	18,088.6 (37,333.8)	86.0	589,141.0
Total population in 2010	1,199.4 (747.2)	2,171.0 (1,162.8)	5,866.5 (2,723.1)	8,259.5 (3,134.1)	7,698.3 (3,016.5)	16,922.0 (9,675.6)	19,227.6 (6,324.4)	29,325.1 (12,402.2)	39,144.6 (24,245.1)	78,418.3 (10,3088.6)	18,654.2 (38,832.7)	75.0	617,594.0
Population change, %	2.5 (10.1)	3.9 (8.9)	(0.9) (7.1)	9.0 (6.0)	5.8 (9.2)	1.4 (6.2)	5.6 (4.1)	2.1 (3.1)	1.4 (3.1)	2.5 (3.3)	3.6 (7.4)	−21.0	33.7
Population change, n	22.2 (115.7)	67.5 (145.2)	(91.3) (362.1)	667.8 (540.3)	388.6 (654.9)	265.5 (1,240.4)	1,042.4 (820.8)	634.1 (841.1)	639.8 (1,273.1)	2,212.2 (5,157.7)	565.6 (1,829.0)	−3,081.0	2,8453.0

Overall high prevalence of obesity and obesity-related unhealthy behaviors indicated the need for community-based wellness interventions in Massachusetts, with average municipal-level obesity estimates exceeding 25%.

Our latent class model yielded excellent classification certainty (Table 2; relative entropy = .995, minimum class probability for any class =.871). In terms of model fit, the Akaike Information Criteria was 80525.5, the Bayesian Information Criteria (BIC) was 82896.0, and the sample size-adjusted BIC was 80948.2. The most populous class contains 51 municipalities (14.5% of the state total), whereas the smallest class contains 20 (5.7%). Model fit statistics did not support the a priori selection of a 10-class solution. The log likelihood was not replicated, and 10 classes were not preferable to 9 classes according to a Vuong-Lo-Mendell-Rubin adjusted likelihood ratio test (*P* = .83). However, a 10-class solution offered the maximum differentiation that program staff could accommodate and was therefore preferred for programmatic reasons.

**Table 2:** Latent Class Membership and Fit in Analysis of Massachusetts Communities

Class	No. of Municipalities Per Class	Percentage of All Municipalities in Class	Mean Class Probability	Minimum Class Probability	Maximum Class Probability
1	49	14.0	0.998	0.959	1
2	31	8.8	0.995	0.941	1
3	26	7.4	0.998	0.970	1
4	44	12.5	0.996	0.855	1
5	36	10.3	0.995	0.921	1
6	51	14.5	0.998	0.969	1
7	40	11.4	0.995	0.871	1
8	20	5.7	1.000	1.000	1
9	21	6.0	0.999	0.992	1
10	33	9.4	0.999	0.989	1

The classification clearly separated the groups of communities from each other. Table 1 shows the mean class-level values of variables across the 10 classes, highlighting the nature of each class and demonstrating the general utility of LCA in characterizing multidimensional complexity.

Class 1 (n = 49) includes Massachusetts’ least densely populated and most rural communities, which tend to have somewhat older and less racially and ethnically diverse residents than the state overall. There are very few retail outlets, physicians, or subsidized housing units in these communities, and the low-density land use patterns contribute to high per household VMT. These municipalities have among the lowest incidence of many negative health outcomes, but indicators of healthy behavior are moderate or poor (eg, childhood obesity, births to mothers who smoked anytime during pregnancy).

In terms of population size and density and geographic location within the state, class 2 is similar to class 1, though with a somewhat more diverse housing stock and a more balanced age structure. Class 2 municipalities struggle with high unemployment, low household incomes, and high poverty rates, and have one of the worst health profiles with low prevalence of fruit and vegetable consumption and physical activity and high prevalence of smoking, births to mothers who smoked during pregnancy, diabetes, and hypertension.

Class 3 is largely composed of moderate-density coastal and western Massachusetts communities (Figure) that are popular seasonal destinations and home to many retirees, as evidenced by the high share of residents aged 65 or older (22%, higher than any other class). Population declined slightly on average in the past decade. Consistent with these land use patterns and demographics, household VMT is lower than in classes 5 and 4, which are of a similar size and density. Health behaviors and outcomes are generally in the middle of the classes, except for hypertension prevalence, which is among the highest in the state.

**Figure:**
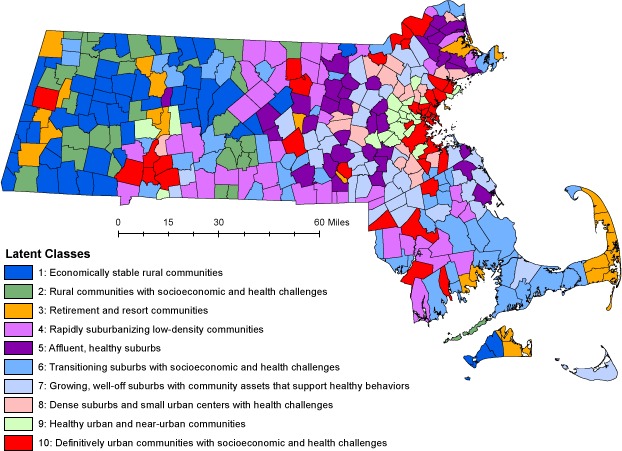
Geographic distribution of latent classes of municipalities in Massachusetts.

Class 4 contains relatively small but rapidly suburbanizing communities with a large share of the population aged less than 18 years. Population in these municipalities grew by 9% on average since 2000, and half the housing stock was built after 1970, mostly in low-density single-family subdivisions. As a result, this is the most car-dependent class, where the average household drives 87 miles per day and more than 90% of workers commute by car. These communities rank near the median on socioeconomic indicators (poverty, income) and health behaviors, but have high rates of hospitalization for cardiovascular and heart disease and transient ischemic attack, or “ministroke.”

Class 5 contains the wealthiest communities in the commonwealth. Median household income is the highest, and poverty rate the lowest of our 10 classes. More than a quarter of the residents are under the age of 18, the highest percentage of young people of any class. These towns also have the lowest childhood obesity rates and the highest rates of physical activity and fruit and vegetable consumption. Class 5 also has among the lowest class-level diabetes prevalence, hypertension diagnoses, and adult obesity.

Class 6 includes a mix of small urban communities and mid-sized suburbs that share numerous socioeconomic and health challenges. Poverty and unemployment rates are well above the state average, household incomes are relatively low, and both health behaviors and outcomes (including smoking, exercise, eating fruits and vegetables, obesity, and hospitalizations) are poor. These communities have among the largest share of residents aged 65 or older, are predominately non-Hispanic white, and have been growing slowly over the past decade.

Class 7 consists of mid-size, moderate density, generally wealthy suburbs in Greater Boston that exhibit generally healthy behaviors and average health outcomes. Compared with other suburban communities, they have a high Asian population (5% average) and a large share of residents aged under 18 (26% average). These communities grew rapidly in the past 10 years yet still have a low share of subsidized housing. Grade 1 and grade 10 obesity rates are among the state’s lowest, as are the rates of adult obesity.

Class 8 has largely mature suburbs and small urban communities in Greater Boston that are characterized by racially and ethnically diverse populations, moderate socioeconomic status, and relatively poor health outcomes. Although they have a relatively diverse housing stock, a large share of the land area is devoted to commercial and industrial uses, mostly automobile-oriented development. It has among the highest class-level hospitalization rates for common chronic diseases, though it falls in the middle of the pack with respect to resident health behaviors.

Class 9 includes a range of higher-density suburbs and compact cities clustered around Boston. This class is characterized by compact development patterns, as indicated by high average population density, a low percentage of commuters traveling by motor vehicle, and a large share of high-density and multifamily housing. In particular, this class has the highest class-level proportion of Asian residents. Class 9 enjoys an excellent health profile, with the lowest adult smoking and obesity rates and highest rates of physical activity and eating fruits and vegetables.

Class 10 is composed of the state’s most populous, densely populated, racially and ethnically diverse, and urban communities. These communities are much less reliant on automobile travel than most other places in the state, but experience substantial socioeconomic challenges, with low incomes, high rates of poverty and unemployment, and a large share of subsidized housing. They perform poorly on almost all health measures. Residents struggle with high rates of physical inactivity, smoking, obesity, and chronic diseases.

## Discussion

We present a novel application of LCA methodology to address programmatic and evaluation challenges associated with implementing a municipal-based wellness intervention program across a large number of heterogeneous communities. The approach considered intervention inputs (eg, retail environment), effect modifiers (eg, age), and outcome measures of the prospective interventions (eg, obesity prevalence). The typology has excellent classification certainty and yielded roughly equal-sized classes. Combining expert local knowledge with an empirical approach was crucial in evaluating face validity and selecting a programmatically useful solution despite model fit statistics. To our knowledge, such an application in community health planning has not been reported in literature.

Although our analysis did not incorporate spatial relationships among municipalities, the typology map reveals that class membership is spatially clustered, reflecting the history of community development, migration patterns, and economic changes in Massachusetts. Land use, transportation, and retail environment indicators clearly distinguished rural, suburban, and urban communities. The level of urban development, however, was not the sole driver of class differentiation. For example, 2 predominantly rural classes (ie, class 1 and class 2) are quite similar with respect to the built environment, housing, and land use characteristics, transportation patterns, the retail environment, and demographics, yet exhibit distinctly different socioeconomic characteristics, health behaviors, and health outcomes. Class 2 communities were poorer and fared worse in overall health profiles, with higher rates of smoking, obesity, hypertension, and hospitalizations for chronic diseases. This distinction informs the design and delivery of public health interventions in those 2 classes.

The prevention and wellness community typology derived from our analysis serves as a basis for 1) establishing proper evaluation benchmarks, 2) establishing efficient-yet-tailored communications campaigns, 3) facilitating knowledge exchange across peer communities, and 4) using cost-effective, context-specific intervention selections and staff training. One of these applications is already in progress: field and telephone survey sample frames used the typology as a stratification variable, ensuring that data on health behaviors, the walking environment, and the food environment are collected on a sample of Mass in Motion and untreated municipalities within each class (T. Land, M. Arcaya, B. Andrews, C. Bartlett, J. Auerbach, K. Kane. A. Pinzon-Marquardt, B. Olendzki, L. Nasuti, W. Li, unpublished data, 2014).

Despite these successes, we note limitations to our analysis. First, the model was constrained to produce 10 classes for pragmatic reasons despite model fit statistics indicating that 2 or more of these classes could be combined. Although we found meaningful distinctions among all 10 groups and made the face validity of the solution our priority, others seeking to apply LCA to public health practice should consider a data-driven approach to obtain a statistically optimal class solution. In making an empirical determination of how many classes exist, technical improvements in model specification, such as relaxing local independence assumptions for highly related variables, should be used. Failing to relax these assumptions may artificially inflate the number of classes detected by LCA, though this was not a major concern in our analysis with a predetermined 10-class solution. Second, although our input variables were selected based on 6 domains that were considered directly relevant to Mass in Motion and CTG programs, it is possible that some inputs are redundant while other relevant indicators were overlooked. Finally, rank-based classification aimed to capture relative rather than absolute similarity among communities and as such served to limit extreme values. For example, the state’s largest community (Boston) has roughly 7,500 times the population of the smallest community (Gosnold). With a decile classification, that ratio is not preserved. Although this methodology limits the impact of extreme values, data from outliers could have been treated in other ways that might have affected the class structure. Absolute similarities may be considered in a refined topology.

LCA, as demonstrated here, is an effective statistical method that could help improve implementation and evaluation of community-based wellness efforts nationwide. Our work in Massachusetts is ongoing. The 5-year evaluation plan uses the latent classes as a fundamental framework for understanding the effect of community interventions; for example, allowing us to examine changes in intervention versus untreated municipalities within a class. If communities across the country aim to make progress combating obesity, smoking, and heart diseases, then targeting the interventions that are more likely to work in specific types of communities will be a positive step forward.

## Appendix. Health, Sociodemographic, and Environmental Input Variables for Latent Class Analysis of Massachusetts Communities

**Table:**

Domain/Short Variable Name	Description	Source
**Health behaviors and outcomes**		
Current smokers (%)	Percentage of adult population aged ≥18 who are current smokers	Massachusetts Behavioral Risk Factor Surveillance System (2009)
Fruit and vegetable consumption (%)	Percentage of adult population aged ≥18 who eat 5 or more servings of fruit and vegetables each day	Massachusetts Behavioral Risk Factor Surveillance System (2009)
Recommended exercise (%)	Percentage of adult population aged ≥18 with no exercise in the past month	Massachusetts Behavioral Risk Factor Surveillance System (2009)
Diabetes (%)	Percentage of adult population aged ≥18 ever been told they have diabetes	Massachusetts Behavioral Risk Factor Surveillance System (2009)
Hypertension (%)	Percentage of adult population aged ≥18 ever been told they have hypertension	Massachusetts Behavioral Risk Factor Surveillance System (2009)
Obese adults (%)	Percentage of adult population aged ≥18 with a body mass index >30	Massachusetts Behavioral Risk Factor Surveillance System (2009)
Births to smokers	Number of births to mothers who smoked at any time during pregnancy per 1,000 births	Massachusetts Registry of Vital Records, births data (2009)
Overweight/obese 1st graders (%)	Percent of 1st graders that are obese or overweight	Massachusetts Department of Public Health School body mass index data (2011)
Overweight/obese 10th graders (%)	Percentage of 10th graders who are obese or overweight	Massachusetts Department of Public Health School body mass index data (2011)
Cardiovascular disease hospitalizations	Unadjusted hospitalization rate for major cardiovascular disease (ICD 390–434, 436–448)	Massachusetts Center for Health Information and Analysis hospital discharge data (2010)
Cerebrovascular hospitalizations	Unadjusted hospitalization rate for cerebrovascular disease (ICD 430–434, 436–438)	Massachusetts Center for Health Information and Analysis hospital discharge data (2010)
Heart disease hospitalizations	Unadjusted hospitalization rate for heart disease (ICD 390–398, 402, 404, 410–429)	Massachusetts Center for Health Information and Analysis hospital discharge data (2010)
Hypertension (and related) hospitalizations	Unadjusted hospitalization rate for hypertension and hypertensive diseases (ICD 401–405)	Massachusetts Center for Health Information and Analysis hospital discharge data (2010)
Stroke hospitalizations	Unadjusted hospitalization rate for transient ischemic attack (ICD 435)	Massachusetts Center for Health Information and Analysis hospital discharge data (2010)
Cardiovascular disease hospitalizations (age-adjusted)	Age-adjusted hospitalization rate for major cardiovascular disease (ICD 390–434, 436–448)	Massachusetts Center for Health Information and Analysis hospital discharge data (2010)
Cerebrovascular hospitalizations (age-adjusted)	Age-adjusted hospitalization rate for cerebrovascular disease (ICD 430–434, 436–438)	Massachusetts Center for Health Information and Analysis hospital discharge data (2010)
Heart disease hospitalizations (age-adjusted)	Age-adjusted hospitalization rate for heart disease (ICD 390–398, 402, 404, 410–429)	Massachusetts Center for Health Information and Analysis hospital discharge data (2010)
Hypertension (and related) hospitalizations (age-adjusted)	Age-adjusted hospitalization rate for hypertension and hypertensive diseases (ICD 401–405)	Massachusetts Center for Health Information and Analysis hospital discharge data (2010)
Stroke hospitalizations (age-adjusted)	Age-adjusted hospitalization rate for transient ischemic attack (ICD 435)	Massachusetts Center for Health Information and Analysis hospital discharge data (2010)
**Built environment, housing, and land use characteristics**		
Population density	Population density (residents per total acres)	US Census (2000), MassGIS land use data (1999)
Housing unit density	Housing unit density (units per residential acre)	US Census (2000), MassGIS land use data (1999)
High-density housing (%)	Percentage of residential land coded as multi-family and high-density (>4 units per acre)	MassGIS land use data (1999)
Medium-density housing (%)	Percentage of residential land coded as medium-density (2–4 units per acre)	MassGIS land use data (1999)
Low-density housing (%)	Percentage of residential land coded as low-density (<2 units per acre)	MassGIS land use data (1999)
Single family homes (%)	Percentage of housing units that are single-family detached homes	US Census (2000)
Developed land (%)	Percentage of land in developed uses	MassGIS land use data (1999)
Vacant developable land (%)	Percentage of land currently undeveloped and not constrained by environmental resources or protected open space	Massachusetts Executive Office of Energy and Environmental Affairs build-out analysis (2001)
Built-out land (%)	Developed land area as a percentage of all unconstrained land area	Massachusetts Executive Office of Energy and Environmental Affairs build-out analysis (2001)
Pre-1939 housing (%)	Percentage of housing units built before 1939	US Census (2000)
Post-war Housing (%)	Percentage of housing units built 1940–1970	US Census (2000)
Post-1970 housing (%)	Percentage of housing units built 1970–2000	US Census (2000)
Sewer accessible housing	Percentage of housing units on public sewer	US Census (1990)
Commercial and industrial	Commercial and industrial land and developed acres	MassGIS land use data (1999)
% Subsidized housing inventory	Percentage of housing units listed on the Subsidized Housing Inventory	Massachusetts Department of Housing and Community Development (2012)
Residential land use change	Percentage change in residential land use acreage, 1971–1999	MassGIS land use data (1971, 1999)
**Transportation patterns**		
Daily vehicle miles traveled	Passenger vehicle miles traveled per household, 2007–2008	MassGIS analysis based on Massachusetts Registry of Motor Vehicles vehicle inspection records
Car/truck/van commuters (%)	Percentage of commuters who commute by car, truck, or van	American Community Survey (2006–2010)
**Retail environment**		
Businesses	Number of establishments	InfoGroup (2011)
Tobacco retailers	Number of tobacco retailers	Massachusetts Tobacco Cessation and Prevention Program Retail Data Management System (fiscal year 2013)
Tobacco retailers selling alcohol	Number of tobacco retailers also selling alcohol	Massachusetts Tobacco Cessation and Prevention Program Retail Data Management System (fiscal year 2013)
Physician count	Number of physicians	Massachusetts Board of Registration in Medicine directory (2009)
**Socioeconomics**		
Unemployment (2000)	Percent unemployment rate, 2000	US Bureau of Labor Statistics, Local Area Unemployment Statistics
Unemployment (2010)	Percent unemployment rate, 2010	US Bureau of Labor Statistics, Local Area Unemployment Statistics
Median household income	Median household income in the past 12 months (in 2010 inflation-adjusted dollars)	American Community Survey (2006–2010)
Poverty rate	Percentage living below federal poverty guidelines	American Community Survey (2006–2010)
**Demographics**		
White (%)	Percentage of the population that is non-Hispanic white race	US Census (2010)
Black (%)	Percentage of the population that is non-Hispanic black race	US Census (2010)
Asian (%)	Percentage of the population that is non-Hispanic Asian race	US Census (2010)
Latino (%)	Percentage of the population that is Hispanic or Latino	US Census (2010)
<18 (%)	Percentage of the population aged <18 years	US Census (2010)
≥65 (%)	Percentage of the population aged ≥65	US Census (2010)
Population, 2000	Population in 2000	US Census (2000)
Population, 2010	Population in 2010	US Census (2010)
Population change (%)	Percent change in population 2000 to 2010	US Census (2000 and 2010)
Population change (count)	Difference in population 2000 to 2010	US Census (2000 and 2010)

Abbreviation: ICD, *International Classification of Diseases*.
